# Resilience in Adverse Contexts: Youth and Clinician Perspectives on Navigating Community Violence

**DOI:** 10.3390/children13010122

**Published:** 2026-01-14

**Authors:** Angel Boulware, Deidra Bibbs

**Affiliations:** 1 Department of Public Health, Johns Hopkins University, Baltimore, MD 21218, USA; 2 Department of Psychiatry, University of Chicago, Chicago, IL 60637, USA; deidralashawn@gmail.com

**Keywords:** community violence exposure, resilience, black youth, mental health

## Abstract

**Highlights:**

**What are the main findings?**

- Resilience among Black youth emerged as a context-specific response to chronic community violence, where behaviors such as hypervigilance and avoidance served protective functions within structurally unsafe environments.
- Clinicians’ work with youth exposed to community violence illuminated how ongoing structural and environmental threats shape mental health, revealing the need for trauma-informed training and institutional practices that reflect these systemic realities.

**What are the implications of the main findings?**

- Trauma-informed care must extend beyond individual treatment to address the structural and environmental conditions that perpetuate community violence, integrating ecological frameworks into clinical practice, community programming, and workforce training.
- Policies and interventions should target the structural determinants that sustain community violence—such as disinvestment, racial inequity, and limited access to mental health resources—by investing in community infrastructure, prevention efforts, and equity-centered trauma services.

**Abstract:**

**Background/Objectives:** Community violence remains a pervasive public health challenge that disproportionately affects Black youth, with lasting impacts on physical and mental health. Traditional models often conceptualize resilience as individual “bounce back” capacity, overlooking how adaptation unfolds amid chronic violence and structural inequity. This study examined how Black youth and trauma clinicians understand, navigate, and redefine resilience within contexts of ongoing community violence exposure. **Methods:** Using a phenomenological qualitative design, the study drew on semi-structured interviews and focus groups with Black youth and clinicians participating in a community violence trauma recovery program in Chicago, Illinois. Data were analyzed thematically to identify patterns in how resilience was described, practiced, and supported. **Results:** Black youth redefined resilience through adaptive survival strategies—such as hypervigilance, avoidance, and emotional regulation—that functioned as protective responses to continuous threat. Clinicians recognized resilience as relational and context-dependent but reported limited training to address trauma rooted in chronic, community-level conditions. Both groups highlighted the role of collective and structural supports, including family, peers, and community networks, in sustaining adaptation. **Conclusions:** Findings highlight the need to expand trauma-informed care beyond individual treatment to address structural conditions that perpetuate community violence. Integrating ecological and culturally grounded models of resilience into clinical training and community programming can improve support for Black youth navigating chronic exposure to violence.

## 1. Introduction

In neighborhoods exposed to community violence, Black youth develop complex strategies to navigate persistent threats, yet these practices are frequently misinterpreted—either celebrated as resilience or pathologized as maladaptive. Community violence remains a pervasive public health challenge with significant impacts on physical, emotional, and social well-being [1,2,3,4]. It is best understood as an outcome of structural violence: the cumulative effects of racial segregation, economic disinvestment, and systemic inequities that heighten the likelihood of interpersonal harm in community settings [5,6]. Defined as exposure to intentional violence outside of close relationships, community violence—whether directly experienced, witnessed, or learned about—disrupts fundamental expectations of safety and stability and, in deeply disenfranchised neighborhoods, constitutes a sustained traumatic environment [3,5].

A substantial body of research demonstrates that exposure to community violence is associated with heightened symptoms of post-traumatic stress, depression, anxiety, and difficulties with emotional regulation [6,7]. Beyond psychological distress, violence influences social functioning, school engagement, perceptions of safety, and trust in institutions, contributing to chronic stress and constrained developmental trajectories [8,9]. These effects often accumulate over time in neighborhoods facing persistent structural disadvantage, where violence is experienced not as an isolated incident but as an ongoing environmental condition.

Yet in many neighborhoods where violence is chronic, residents continue to adapt, support one another, and sustain daily life despite ongoing threats [10]. These patterns highlight the importance of examining not only the harms of violence but also the adaptive processes that enable individuals and communities to navigate ongoing adversity. This shift—from documenting the consequences of violence to understanding the strategies youth develop in response introduces resilience as a critical, and often contentious, concept within contexts of chronic community violence.

Understanding these adaptive processes requires moving beyond individualistic notions of resilience toward frameworks that recognize the structural, cultural, and collective conditions under which resilience develops. Such frameworks foreground the reality that youth and families are navigating environments shaped by structural inequity, limited institutional protection, and persistent exposure to threat [11].

This research investigates the everyday practices Black youth use to navigate community violence and argues that these strategic responses—shaped by structural constraints—are routinely misread within dominant narratives of resilience, either valorized as resilience or dismissed as maladaptive.

The current study contributes to this growing body of work on resilience and exposure to community violence by exploring the experiences of Black youth and the clinicians who support them. Drawing on semi-structured interviews and a youth focus group, this analysis illuminates how young people and providers understand, practice, and negotiate resilience in contexts marked by chronic community violence. The study examines not only the strategies youth employ to manage stress and maintain well-being, but also the structural constraints and social dynamics that shape those strategies. Through the perspectives of both youth and clinicians, this work advances a more contextualized understanding of community violence exposure and its effects on young people. It also offers a culturally grounded account of adaptation, highlighting the relational, communal, and structural processes that enable resilience and, at times, complicate it—within high-violence environments. In doing so, the study deepens both theoretical and practical insights into how resilience is navigated, challenged, and redefined in the context of community violence.

### Literature Review

To situate this study within existing scholarship, this review synthesizes research on community violence exposure, coping, and conceptualizations of resilience—particularly as they pertain to Black youth navigating structurally marginalized environments.

The American Psychological Association defines resilience as “the process of adapting well in the face of adversity, trauma, tragedy, threats, or significant sources of stress” [11,12]. Youth with greater access to supportive relationships, coping resources, and community cohesion tend to experience fewer trauma symptoms and more stable emotional and social functioning [13,14]. Conversely, limited access to safe environments and responsive institutions can undermine resilience and intensify chronic stress.

Building on this definition, scholars have challenged narrow, individual-focused interpretations of resilience. Once described as the capacity to “bounce back,” resilience is now understood as a dynamic, context-dependent process shaped by social relationships and environmental conditions [11,15]. In communities experiencing chronic stressors such as persistent violence exposure, adaptation rarely involves returning to a prior baseline. Instead, it reflects ongoing negotiation with structural constraints that shape both the capacity for and pathways of recovery [5,16,17]. These perspectives underscore the need to examine resilience within its broader social and structural context.

These frameworks are especially critical for understanding how resilience operates among Black youth, who face disproportionate exposure to community violence. In 2022, Black youth accounted for 48% of all youth firearm deaths despite representing only 14% of the U.S. youth population [18,19]. Repeated exposure to violence and neighborhood disadvantage increases risk for post-traumatic stress, depression, and anxiety [20] while restricting opportunities for recovery. Yet many Black youth demonstrate considerable resilience, drawing on cultural strengths, community networks, and survival strategies shaped by their lived environments [21,22]. However, dominant conceptualizations of resilience have not always reflected these realities. Much of the existing research still emphasizes individual traits or outcomes—such as coping skills or academic achievement—while overlooking the collective, cultural, and structural conditions that shape adaptation. As a result, prior work has often failed to capture the nuanced ways Black youth navigate adversity within contexts characterized by chronic threat and limited institutional support.

Furthermore, few studies have examined how racism, historical trauma, and chronic exposure to violence influence both the need for and expression of resilience, or how youth define and sustain it within systems of inequity [21,23]. Emerging ecological frameworks attempt to address these limitations by conceptualizing resilience as “a dynamic process of transactions within and among multiple levels of children’s environments over time” [24]. These models move beyond trait-based perspectives by emphasizing the interplay between individual, family, community, and structural factors. Yet there remains limited research on how such ecological models apply to contexts of chronic community violence, particularly for populations disproportionately affected by structural inequities.

Against this conceptual backdrop, empirical studies illustrate how community violence shapes emotional, cognitive, and behavioral responses among youth. Exposure may involve direct victimization, witnessing violence, or residing in high-crime neighborhoods—all of which negatively affect mental health [2,4,25]. Neighborhood-level conditions such as concentrated violent crime further influence academic performance, emotional well-being, and perceived safety [26]. These harms are compounded by poverty, racism, and limited access to health and educational resources, which collectively weaken protective systems [17,27].

Within these environments, behaviors often labeled maladaptive may function as meaningful adaptive strategies. Chronic exposure to violence is associated with hypervigilance, emotional dysregulation, and avoidance—responses that can serve as survival mechanisms in threatening contexts [28,29,30,31,32]. For example, Smith and Patton demonstrated how Black youth in Baltimore used hypervigilance and avoidance to navigate daily dangers, challenging dominant notions of “maladaptive” coping [26,33]. These strategies may reduce exposure to overwhelming cues [20,34] and reflect community-specific adaptations to structural oppression [17,31]

Concurrently, youth coping unfolds within broader systems of support. Families, peers, schools, and community networks play critical roles in buffering psychological harm and promoting adaptive functioning [35,36,37,38]. When community-level stressors such as disinvestment or inequitable access to health care are addressed, youth outcomes improve [35,38,39]. Yet chronic violence complicates recovery, as youth must continually adapt rather than resolve discrete traumatic events [2,40].

Despite these constraints, many Black youth demonstrate sustained adaptive strength, even though resilience may come with psychological costs. Recognizing these dual realities—persistent vulnerability alongside sustained adaptation—has informed the development of community-based initiatives such as Community Violence Intervention (CVI) and Hospital-Based Violence Intervention Programs (HVIPs), which aim to disrupt cycles of violence and promote youth healing [41,42,43]. Yet evaluations show persistent gaps in trauma-informed care, coordination, and attention to youths’ psychosocial realities [44].

Clinical interventions further highlight both possibilities and limitations in existing approaches to supporting youth exposed to chronic violence. Cognitive-behavioral evidence-based treatments such as Trauma-Focused Cognitive Behavioral Therapy (TF-CBT) are among the most empirically supported interventions for trauma-exposed youth [38,41]. School-based adaptations such as Cognitive Behavioral Intervention for Trauma in Schools (CBITS) have also shown reductions in PTSD and depressive symptoms among urban youth [45], and group-based models such as Structured Psychotherapy for Adolescents Responding to Chronic Stress (SPARCS) target affect regulation, interpersonal effectiveness, and meaning-making [46]. However, a notable gap remains. For example, while TF-CBT is well validated for discrete traumatic events, far fewer trials have examined its adaptation for chronic community violence. Most empirical evidence comes from school-based CBITS rather than caregiver-inclusive TF-CBT models. Recent reviews affirm the general efficacy of CBT and TF-CBT but highlight the need for context-specific adaptations in chronically violent and disinvested communities [45]. Practical adaptations conceptualize trauma as both past and ongoing rather than a singular event [47] and integrate culturally responsive approaches that account for race, culture, and systemic inequality [48].

These limitations highlight the need for research that centers on youth perspectives and attends to how resilience is lived rather than merely measured. While some youth maintain positive developmental trajectories despite adversity [4], celebrating resilience without acknowledging the cumulative toll of constant adaptation risks obscuring long-term strain. Continuous recovery demands may elevate allostatic load, contributing to chronic physiological wear [4]. Resilience is also domain-specific; youth may excel academically while struggling emotionally [49,50].

Taken together, this body of research emphasizes that resilience is not static or universally positive but a socially and contextually embedded process shaped by intersecting systems of inequality. However, much of the existing literature relies on standardized measures of resilience that capture outcomes or trait-like characteristics but do not illuminate how youth themselves understand, interpret, and enact resilience in conditions of chronic threat. Quantitative measures and intervention evaluations rarely capture the lived complexities, meaning-making processes, and tensions youth navigate daily.

What remains missing—and what motivates the present study—is an in-depth, phenomenological examination of how Black youth and clinicians conceptualize and practice resilience amid ongoing community violence. A phenomenological approach is uniquely suited to reveal the subjective, relational, and structural dimensions of resilience that remain obscured in studies focused solely on symptoms, treatment outcomes, or individual coping skills.

The current study examines resilience as both a social label and a lived concept by drawing on the perspectives of Black youth and trauma clinicians within a community violence trauma recovery program. Although resilience scholarship increasingly acknowledges the role of social and structural context, less attention has been given to how resilience is interpreted and enacted under conditions of chronic violence and systemic neglect. In these contexts, adaptive strategies may not align with dominant or normative resilience frameworks. By incorporating the perspectives of both youth and clinicians, this study aims to illuminate how resilience is understood, negotiated, and expressed in practice, offering a more contextually grounded account of resilience among Black youth exposed to ongoing community violence.

## 2. Materials and Methods

### 2.1. Study Design

This study used a phenomenological qualitative design to explore the lived experiences of Black youth affected by community violence and the clinicians providing trauma-informed care within a community violence trauma recovery program. Phenomenology was chosen for its focus on how individuals interpret and make meaning of their direct experiences [51,52,53]. This approach reflects the study’s commitment to centering youth voices as they navigate life during and after violence exposure, while also considering how clinicians’ professional experiences shape their interpretations of resilience and recovery. Consistent with guidance on rigor in interview-based research [54], the study design emphasized transparency in sampling, data collection, and analytic decision-making.

By reviewing both youth and clinician perspectives, the study sought a deeper, contextually grounded understanding of trauma-informed care and resilience in under-resourced urban communities disproportionately affected by violence. Rather than imposing preconceived frameworks, phenomenology allowed themes to emerge inductively from participant narratives. Data were collected through semi-structured interviews, which enabled participants to share their experiences in their own words while allowing for flexibility in follow-up questions [55]. This method preserved the richness of individual experiences while identifying broader patterns in how resilience is constructed and sustained in the face of chronic adversity.

### 2.2. Research Site

Data were collected from a community violence trauma recovery program based at a hospital in Chicago, Illinois, which serves youth disproportionately impacted by violence. The program offers trauma-informed care and support to individuals regardless of injury status. An interdisciplinary outpatient clinic within the program provides brief interventions, psychological and psychiatric assessments, and referrals to ongoing therapy for youth exposed to violence. The hospital alerts the clinic when pediatric patients are admitted due to violent injury, prompting outreach to caregivers. The clinic also accepts referrals from other healthcare providers. While participation in the broader trauma recovery program includes the clinic, the clinic and the program are mutually inclusive. The clinic additionally trains clinicians to deliver trauma-informed care tailored to underserved urban populations.

Many of the families served face intersecting social determinants, including economic instability, housing insecurity, and limited resources. These challenges complicate recruitment and retention, requiring the clinic’s team to engage in repeated outreach and flexible scheduling to support and connect with people.

### 2.3. Participant Recruitment

This study used convenience sampling to recruit participants from a community violence trauma recovery program [56]. The co-director of the program’s clinic generated a list of eligible youth and clinicians. To ensure participant safety, individuals experiencing acute crisis or extreme trauma were excluded.

Youth were eligible if they (a) had previously engaged with the trauma recovery program or clinic (defined as completing at least one clinical intake or treatment session), (b) were between 15 and 20 years of age, and (c) were not in acute crisis at the time of recruitment. Clinicians were eligible if they were current or former employees or trainees at the clinic. In alignment with the clinic’s service criteria, all eligible participants had prior exposure to community violence.

Recruitment was conducted via telephone outreach, and interviews were completed virtually. Thirty youth were contacted; nine completed interviews. Eight initially agreed but were unable to complete participation due to circumstances including family emergencies, housing instability, or incarceration, and did not respond after three follow-up attempts. Thirteen youth did not respond or had outdated contact information. Youth varied in their level of program involvement, ranging from initial assessments to ongoing therapy; to minimize bias, information regarding treatment level or clinician assignment was not shared with the research team.

Of eight eligible clinicians, six completed interviews; two declined participation due to scheduling conflicts. Recruitment continued until thematic saturation was reached, defined as the point at which no new codes or patterns emerged in later interviews [51,57,58].

### 2.4. Participants

The final sample consisted of 15 participants: nine youth (*n* = 9) and six clinicians (*n* = 6), all affiliated with the same community violence trauma recovery program. All participants were assigned pseudonyms to protect confidentiality.

Among clinician participants, two were former staff or trainees, and four were currently employed by the clinic at the time of the study. The clinician group included two clinical psychology interns, two licensed clinical psychologists, one postdoctoral fellow, and one mental health counselor—four clinicians identified as Black, one as White, and one as Latino. Five clinicians were identified as female and one as male. (see Table 1).

Youth participants ranged in age from 15 to 20 years (M = 17.67, SD = 2.18). All youth identified as Black, reflecting both the demographic composition of the clinic’s service population and the study’s focus on the experiences of Black youth exposed to community violence in Chicago (See Table 2).

### 2.5. Procedure

After obtaining Institutional Review Board approval on 2 October 2020, participants were recruited via convenience sampling. Interviews and the focus group were conducted via Zoom. For youth under age 18, verbal consent from a parent or guardian and verbal assent from the youth were obtained prior to participation. The IRB approved the use of verbal consent and assent to facilitate remote data collection and minimize participant burden.

Interview Protocol: Participants took part in semi-structured interviews designed to facilitate open dialogue [59]. The interview guide was developed based on observational data, relevant resilience literature [21], and select items from the Connor-Davidson Resilience Scale [60]. Interviews were guided by broad, open-ended questions about participants’ daily environments, sources of stress, coping strategies, and sources of support. The guide did not directly ask youth to disclose specific traumatic or violent events; however, experiences related to community violence emerged organically as participants described their neighborhood context and daily challenges. A list of the core topic areas included in the interview guide is provided in Appendix A.

Clinician interviews focused on their applied psychosocial understanding of resilience, survival, and coping from the perspective of mental health providers at the site. Youth interviews centered on how participants have responded to and coped with community violence and intersecting traumas, rather than on the details of the traumatic events themselves. Following the interviews, the youth were invited to participate in a focus group to discuss emerging themes collectively and reflect on shared experiences of community violence.

Focus Group Protocol: Preliminary themes identified during individual youth interviews informed the development of a semi-structured focus group guide. A 90-min focus group was conducted with six youth participants. The session fostered peer dialogue and collective reflection, allowing the research team to explore how social dynamics and group meaning-making shape coping and resilience-making. In contrast to the one-on-one interviews, which captured individualized narratives and personal coping strategies, the focus group illuminated the relational and communal aspects of resilience, including the “social costs” that emerge in group or peer contexts.

### 2.6. Analysis

A mixed-methods analytical approach combining inductive and deductive coding was used, reflective thematic analysis (RTA) within an inductive-deductive framework. Drawing on Saldaña’s methodology [61], deductive coding initially sorted the data into broad thematic categories aligned with the research questions and interview protocol. This phase was based on sensitizing concepts drawn from theoretical frameworks and the study’s aims. Inductive coding then identified additional themes emerging from the data beyond these predefined categories, allowing discovery of patterns, nuances, and unique insights [62].

This combined approach provided a structured yet flexible framework that deepened understanding of participants’ experiences with community violence [63,64]. Because the focus group captured interactive, peer-based meaning-making that differed from the individualized narratives of the interviews, these two data sources were first coded separately to preserve their distinct contributions. The research team then used a triangulation strategy to compare patterns across the interviews and the focus group, examining how themes were reinforced, expanded, or questioned when youth engaged in collective dialogue. This process strengthened the credibility of the findings by grounding interpretations in both individual and group-level perspectives.

Analysis was conducted using Dedoose software 9.0. A codebook was developed to organize prominent themes from interviews and focus group discussions. To ensure validity and reliability, all research team members independently reviewed the codebook and provided feedback to refine and finalize the thematic categories. Consistent with a reflexive thematic analytic approach, multiple team members independently coded a subset of transcripts and engaged in iterative, reflexive discussion to examine divergent interpretations and refine analytic decisions, rather than to calculate statistical inter-rater reliability [61,65].

A primary goal was to synthesize themes across clinician and youth interviews and the youth focus group to identify key patterns in participants’ experiences and understandings of community violence and resilience.

### 2.7. Researcher Positionality

The first author of this study became affiliated with the community violence trauma recovery clinic while serving as a study coordinator, where they led the assessment and evaluation of the clinic’s treatment model, focusing on enhancing services for families affected by community violence. In this role, the author also managed multiple research initiatives, including a feasibility study that gathered feedback from patients, caregivers, and clinicians through focus groups, surveys, and community advisory board meetings. Through this work, the author engaged directly with families, staff, and clinicians, gaining nuanced insight into the complex challenges youth and families navigate within the clinic setting.

The second author of this study became affiliated with the community violence trauma recovery clinic during their postdoctoral fellowship, after data collection for this study, and served as one of the clinic’s primary clinicians. In this role, the author provided trauma-focused psychological assessments, psychotherapy, and case management to children and adolescents impacted by community violence. In addition to their clinical responsibilities, the author contributed to ongoing research efforts within the clinic, offering a unique perspective grounded in direct therapeutic engagement with youth and families. This dual role enabled the author to integrate clinical insight into the research process, particularly in identifying trauma-related needs, barriers to care, and culturally responsive approaches to healing in high-risk community contexts. Importantly, the author also brings personal insight to this work, having grown up in and around the same communities served by the clinic. This lived experience, combined with clinical expertise, deepens the author’s commitment to honoring the voices and realities of the youth and families engaged in this project.

Some participants in the present study were individuals whom the authors had previously encountered during their time at the clinic. The authors’ earlier work and this project are distinct, with no overlap in timeline or study objectives. Although participants were recruited from the clinic’s programming, their involvement in the study was entirely voluntary, and no prior relationship with the author influenced their decision to participate.

## 3. Results

### 3.1. Findings

The findings of this study illustrate how Black youth and trauma clinicians conceptualize, negotiate, and critique resilience in the context of chronic community violence. Although clinicians and youth often described overlapping experiences of survival and adaptation, they attached different meanings, expectations, and emotional burdens to resilience as both a construct and a lived reality. Rather than functioning as a stable trait or outcome, resilience emerged as a contested concept—one that youth often resisted, and clinicians frequently questioned—revealing the structural, relational, and psychological tensions that shape adaptation in high-violence environments.

To guide interpretation, the themes below reflect not only what participants reported but also how they made meaning of resilience, including the ways they challenged dominant psychological definitions, exposed the limits of clinical training, and articulated the high personal and social costs of being labeled “resilient.”

#### 3.1.1. Theme 1: Divergent Understandings of Resilience

This theme captures how youth and clinicians expressed different levels of familiarity, acceptance, and critique of resilience. While youth focused on how the label was externally applied, often inaccurately, clinicians concentrate on the concept itself, questioning its cultural grounding, structural assumptions, and clinical utility.

Many clinicians described a growing discomfort with the term “resilience”, noting that its widespread use in psychology can obscure the structural conditions that necessitate constant adaptation. Rather than understanding resilience as an empowering construct, clinicians viewed it as a concept that risks shifting responsibility for survival onto individuals while ignoring the systemic and racialized conditions that produce harm.

“I don’t like the word resilience. My field loves it. White psychology loves the construct of resilience. To me, it means making do with an unjust life, surviving being here, and appearing whole in the context of injustice.”

—Dr. Briana

For youth, the term “resilience” was largely unfamiliar or felt disconnected from their lived experiences. They often experienced it as a label used by others, including schools, programs, clinicians, but rarely used it to describe themselves. When youth were labeled “resilient”, the designation came with emotional expectations that felt unachievable or unfair.

Jaylen described receiving a resilience award after returning to school following a shooting. Instead of feeling honored, he felt pressure to maintain an image of strength:

“Sometimes I feel like it’s a lot of pressure. Because I get called many things, but at times, I know I break down a lot. It’s kind of like being a leader of something like a group and letting them see that you’re weak or that there’s something that’s affecting you, and it’s just like so much pressure for me... I survived things of that nature, but it’s the after-effects.”

—Jaylen, 20-year-old male

Youth’s resistance to the term reveals a key analytic insight: resilience functioned less as a lived identity and more as an imposed narrative that obscured ongoing distress. These findings challenge conventional resilience frameworks, which often assume that individuals recognize or internalize resilience as a positive attribute.

Together, youth and clinicians’ critiques of the concept of resilience reveal that the term itself is insufficient for capturing the lived realities of chronic violence. These tensions set the stage for the next theme, which shows that for participants, resilience begins not with achievement or emotional recovery, but with the basic act of surviving ongoing threat or harm.

#### 3.1.2. Theme 2: Survival as the Foundation of Resilience

Both clinicians and youth described survival—not personal growth or thriving—as the most immediate and meaningful expression of resilience. Survival was not framed as a precursor to resilience but as its core: in contexts marked by chronic threat, staying alive is resilience.

“I think kids just being able to survive is a big win. Unfortunately, it’s sad to say, but kids being able to survive these environments is resilience.”

—Dr. Mario.

“Sometimes, surviving is resilience. Sometimes it is just breathing, being alive.”

—Dr. Briana.

Clinicians emphasized the instinctual, urgent nature of survival strategies:

“Things that you have to employ right away are linked with high levels of distress… because you might die.”

—Dr. Mario.

Youth articulated similar strategies in concrete terms—avoiding certain places, staying indoors, or moving through the neighborhood with heightened vigilance:

“Just don’t stay out too late… If you want to survive, you’ve got to move, as nobody can touch you. Like a president.”

—Cameron, 19-year-old male.

Analytically, this theme emphasizes that what clinical models often categorize as “maladaptive “coping—avoidance, hypervigilance, emotional numbing—can be essential strategies for navigating structurally unsafe environments. Both groups recognized that survival consumes psychological energy and limits opportunities for emotional recovery, highlighting how structural violence shapes the boundaries of what resilience can realistically look like.

While survival emerged as the foundation of resilience, participants also highlighted that survival alone is not static—over time, these urgent protective behaviors evolve into long-term adaptations. The next theme explores this progression, illustrating how survival strategies become embedded in daily life amid chronic and unchanging environmental danger.

#### 3.1.3. Theme 3: Adaptation as a Continuous, Dynamic Process

Participants described resilience as a long-term process in which short-term survival strategies become entrenched adaptations. This progression reflects a central challenge of community violence: recovery is difficult when the environment remains dangerous.

Clinicians articulated this tension clearly:

“This is what makes trauma recovery so challenging, especially if your environment never changes… You have me, as your therapist, talking to you about hypervigilance and the stress and strain it puts on your body. But it is also adaptive because when the world you live in on a day-to-day basis is potentially threatening all the time, you’re ready to protect yourself. That’s adapting.”

—Dr. Briana.

Youth similarly described adapting as “getting used to” ongoing violence—not because it becomes less harmful, but because adaptation becomes necessary:

“I wouldn’t really say adapting to it… I’m getting used to it because I’ve been living over here for so long… Like, there’s no more really adapting that I can do, at least living over here.”

—Kyla, 15-year-old female.

Clinicians differentiated between adapting and adapting well. The term “adapting” reflects a minimal, survival-based adjustment, essentially becoming accustomed to difficult or unsafe circumstances. In contrast, adapting well denotes a qualitatively different process that reflects positive coping, growth, and thriving despite chronic exposure to adversity:

“Adapting well is... acknowledging that this is the reality… but actively being involved in things that balance out this harmful reality.”

—Dr. Mario.

Youth provided their own definitions of what it means to adapt well. Cameron focused on health and activity:

“Well, I eat better than I used to, for sure. I like to exercise… It still needs more healing, but I have to stay active.”

—Cameron, 19-year-old male.

Jaylen defined adapting well as regaining a sense of normalcy and independence:

“Being comfortable with any activity, being able to go outside as freely as I used to. “

—Jaylen, 20-year-old male.

He also reflected on how others might fail to adapt well, such as a peer who sought violent revenge after being injured:

“Going for revenge and vengeance is easy. The hard part is taking the acceptance of life for what it is and making the best of it.”

—Jaylen, 20-year-old male.

Youth’s definitions of “adapting well” reveal how adaptation is negotiated within the boundaries of what is structurally possible.

Yet even as youth and clinicians described adaptation as necessary and ongoing, they also emphasized that adapting comes with significant personal and social burdens. The following theme examines these costs, revealing how the expectation to continually withstand adversity creates emotional strain and shapes relationships, identity, and perceptions of strength.

#### 3.1.4. Theme 4: The Emotional and Social Costs of “Being Resilient”

Across interviews, youth and clinicians viewed resilience as a burden, an expectation that requires emotional suppression, independence, and constant strength. Youth consistently described resilience as something demanded of them rather than something they chose. These perceptions emerged consistently across individual interviews and were reinforced in focus group discussions.

Jaylen shared how being perceived as resilient created pressure to appear unaffected:

“The problem with this is when people see you as an uppity person with positive energy, and they finally see you down, it can come off weird to them because they see you as being a positive person who does not let things get them down.”

—Jaylen, 20-year-old male.

While these themes were prominent in individual interviews, focus group discussions uniquely highlighted the perceived benefits of resilience, particularly its strategic value in navigating social and institutional contexts. Youth also described how being resilient sometimes created tension in their social circles. Trey emphasized that the resistance he encountered came not from himself, but from others who were uncomfortable with his growth or change.

“It is not common that people want you to win. Many people want you to stay down with them and don’t want you to change your behavior. Even your own family.”

—Trey, 20-year-old male.

The focus group collectively acknowledged that the meaning and consequences of resilience shift depending on who perceives it, whether family, clinicians, or peers. In clinical settings, resilience may lead to assumptions that youth do not need help. In peer or community contexts, resilience may be a requirement for survival.

This theme highlights an important theoretical insight: resilience can function as a social expectation that obscures suffering and discourages help-seeking, particularly in clinical contexts where perceived resilience may lead providers to underestimate distress.

Together, these themes demonstrate that resilience in contexts of chronic community violence is neither a fixed trait nor an uncomplicated strength; rather, it is a contested, burdensome, and structurally constrained process shaped by survival, long-term adaptation, and the social pressures to appear unaffected by ongoing harm.

## 4. Discussion

This study explored the strategies Black youth use to navigate exposure to community violence and how clinicians interpret these behaviors—at times identifying them as resilience, and at other times recognizing them as context-driven adaptations that challenge conventional resilience paradigms. Guided by the study’s research questions, the findings show that resilience in high-violence environments is shaped by survival, continuous adaptation, and the social pressures surrounding strength and vulnerability. Rather than reflecting a stable personal trait, resilience emerged as a dynamic, relational, and structurally constrained process marked by competing meanings between youth and clinicians.

### 4.1. Disconnected Understandings of Resilience

A central finding is the disconnection between traditional psychological definitions of resilience and the lived realities of Black youth navigating chronic community violence. Clinicians recognized that dominant “bounce-back” models do not translate well in contexts where traumatic events are continuous rather than isolated. Their critiques resonate with ecological resilience frameworks that emphasize how structural racism, persistent neighborhood danger, and limited institutional support shape young people’s capacity to cope [21,24]

Youth, in contrast, rarely identified with the term “resilience”. Many described the label as externally imposed—a narrative that pressured them to appear recovered even when they still felt distressed. Their responses illustrate how resilience can become a burden, obscuring ongoing needs and discouraging expressions of vulnerability. This disconnect suggests that resilience is not simply an internal characteristic but is shaped by social expectations, institutional narratives, and the conditions required for emotional survival. Clinically, this highlights the need for practitioners to rely less on resilience rhetoric and more on youth-defined language that captures their lived experiences. These findings align with critiques of resilience frameworks that prioritize emotional regulation and symptom reduction, which may inadequately capture adaptation under conditions of chronic adversity [66,67].

### 4.2. Survival as the Foundation of Resilience

Youth and clinicians consistently emphasized survival—rather than growth or thriving—as the core expression of resilience. Strategies such as vigilance, staying indoors, or altering routes through the neighborhood were described as essential tools for staying safe. Clinicians acknowledged that behaviors often pathologized in traditional trauma models can be protective in contexts marked by persistent threat. These interpretations align with research documenting hypervigilance and avoidance as functional responses to chronic community violence [26,68].

This conceptualization contrasts with dominant resilience frameworks in developmental and clinical psychology, which frequently operationalize resilience through indicators such as emotion regulation, symptom reduction, or positive mental health outcomes. In these models, resilience is often inferred from youths’ ability to regulate emotions, cognitively reframe adversity, or demonstrate psychological well-being following discrete stressors.

In the context of chronic community violence, however, these benchmarks may inadequately capture youth adaptation. For participants in this study, resilience was less about emotional recovery or “bouncing back” and more about staying alive, alert, and able to navigate ongoing risk.

These findings are consistent with qualitative research on chronic maltreatment and prolonged adversity, which similarly challenges trait-based or outcome-oriented definitions of resilience. For example, Yoon et al. (2020) demonstrate that youth exposed to sustained maltreatment often describe resilience as endurance and survival rather than recovery or growth, emphasizing functional coping even when psychological distress persists [67].

More recent qualitative work by Green and Meadows (2025) further illustrates how Black people, specifically Black men living under chronic adversity articulate resilience as persistence under constraint, rather than as evidence of resolved trauma or improved mental health [68].

Together, this literature reinforces the idea that resilience under chronic threat may coexist with ongoing distress rather than signal its absence; while Green and Meadows (2025) [68] examine adults, the present findings suggest that similar understandings of resilience are articulated by youth navigating chronic community violence.

Understanding survival as resilience complicates traditional clinical approaches that emphasize emotion regulation or cognitive reframing. In communities where danger is ongoing, survival strategies may be both necessary and exhausting. These findings reinforce arguments that resilience must be understood within environmental constraints: youth are not merely responding to trauma but adapting to ongoing structural conditions. For practitioners, this emphasizes the importance of validating survival strategies and tailoring therapeutic expectations to acknowledge ongoing exposure to harm.

### 4.3. Adaptation as a Continuous, Dynamic Process

The transition from immediate survival behaviors to long-term adaptations further demonstrates that resilience is an iterative process shaped by repeated exposure. Youth described “getting used to” violence, not because it became less harmful, but because adaptation became necessary for navigating daily life. Clinicians echoed this understanding, noting that conventional recovery models that assume a return to baseline functioning may be unrealistic when the environment remains unsafe. These findings echo broader discussions of resilience as a dynamic, context-dependent process, shaped by structural inequities and chronic adversity [11,14,16,69].

This pattern contrasts with other forms of childhood adversity, such as child labor, in which resilience often involves endurance, skill acquisition, or the development of agency amid economic hardship. In the context of community violence, resilience is rooted in physical safety, vigilance, and relational alertness. This comparison highlights that different adversities produce distinct resilience pathways shaped by the nature and immediacy of threat.

Clinically, understanding adaptation as continuous—rather than as an endpoint—invites shifts in therapeutic goals toward supporting ongoing coping, enhancing safety-oriented strategies, and acknowledging the limits of recovery in persistently violent environments.

### 4.4. The Emotional and Social Cost of Performing Resilience

The findings also highlight the emotional and relational burdens associated with being perceived as resilient. Data primarily emerging from the focus group, documented youth describing the need to project strength to avoid being targeted or judged, yet this performance often restricted their ability to seek help or express vulnerability. Within peer networks and even within families, resilience functioned as a social identity that protected them but simultaneously isolated them. Similar dynamics have been documented in qualitative studies of chronically exposed youth, where resilience operates as a socially necessary performance that conceals vulnerability while preserving safety and social standing [67].

These dynamics reveal resilience as both protective and costly. The pressure to appear “okay” may reduce access to support systems, as clinicians or community members misinterpret muted emotional expression as evidence of coping. Recognizing resilience as a performed social strategy challenges practitioners to create therapeutic spaces where vulnerability does not carry interpersonal risk. Programs that explicitly address emotional suppression, peer expectations, and the social meanings of strength may better support youth navigating chronic adversity.

### 4.5. Limitations

This study has several limitations that should be considered when interpreting the findings. First, the sample was drawn from a single community violence trauma recovery clinic, which limits generalizability to other settings or populations. Recruitment challenges such as limited clinic volume, unstable contact information, and competing life demands resulted in a smaller youth sample than anticipated. Nonetheless, thematic saturation was achieved, and the 15 combined interviews with youth and clinicians provided sufficient depth to address the research questions.

Second, clinicians were at varying stages of their careers, including trainees and newly licensed practitioners. While this diversity offered a wide range of perspectives, it may also introduce variability in clinical interpretation and practice.

Third, the phenomenological approach, though well-suited to capturing the nuance of lived experiences with community violence, inherently limits external validity. Phenomenological analysis reflects the meaning-making of a relatively small group and may not capture the full range of experiences among all youth exposed to community violence. To strengthen credibility and reduce analytic bias, particularly given the research team’s prior relationship with the clinic, external coders unaffiliated with the site participated in coding and theme development.

Finally, structural inequities affecting many families served by the clinic created barriers to participation and may have limited the sample’s demographic diversity. These contextual constraints should be considered when evaluating the transferability of the findings to other communities or service settings.

### 4.6. Future Research

Future research should continue exploring how youth navigate and adapt to chronic adversity, using culturally and contextually grounded approaches to inform interventions and programs. Including the perspectives of both youth and clinicians can enrich our understanding and guide the development of more effective and relevant treatments. Although this study draws on a small sample, the findings offer insights that can support broader efforts to improve clinical and community-based interventions for youth exposed to community violence.

## 5. Conclusions

This study examined resilience as experienced by Black youth in violence-affected communities, integrating perspectives from both youth and trauma clinicians. A key strength of this study is that it captures the dynamic interplay between youths’ lived experiences and clinicians’ observations, offering a more holistic understanding of resilience and the impact of exposure to community violence. The findings highlight that resilience in this context is not simply an individual trait or endpoint, but a dynamic process shaped by survival strategies, long-term adaptations, and the social and structural contexts in which youth live.

Survival strategies—employed to stay safe—emerged as a foundational form of resilience, often taking precedence over traditional ideas of personal growth or recovery. Over time, these strategies evolve into adaptive behaviors, reflecting ongoing learning and adjustment to persistent adversity.

The study also highlights the significant emotional and social costs associated with performing resilience. Black youth often feel pressured to appear strong, which can limit their emotional expression and negatively impact relationships, while clinicians must balance supporting adaptive behaviors without overburdening them. Understanding resilience in context is therefore critical because it is both necessary for survival and demanding in its social and emotional consequences.

In conclusion, Black youth are often praised for their resilience or pathologized when their coping strategies are misunderstood or defy conventional norms. However, centering their ability to survive solely on what they endure misses the broader context. Such framing overlooks the structural and social conditions that shape both their challenges and their capacities to adapt. Attention must shift toward understanding the social and structural contexts in which these coping strategies emerge to better support youth in developing healthier, more sustainable responses. Such understanding is essential for designing comprehensive interventions and therapeutic practices that are grounded in youths’ lived realities. This perspective can advance the development of more effective, culturally responsive programs and trauma-informed models of care that reflect the experiences of young people living in high-violence environments.

## Figures and Tables

**Table 1:** Clinician Participants’ Demographics.

Pseudonym	Title	Race/Ethnicity	Gender
Dr. Kara	Clinical Psychologist (PsyD)	Black	Female
Dr. Nicole	Clinical Psychologist (PhD)	White	Female
Dr. Brianna	Postdoctoral Fellow in Clinical Psychology	Black	Female
Nia	Clinical Psychology Intern	Black	Female
Mario	Clinical Psychology Intern	Latino	Male
Amanda	Clinical Mental Health Counselor (LCMHC)	Black	Female

Note. Data were collected from clinician participants (*n* = 6) at the Community Violence Trauma Recovery Program.

**Table 2:** Youth Participants’ Demographics.

Pseudonym	Type of Violence Exposure	Gender	Age
Trey	Direct and Indirect	Male	20
Mya	Indirect	Female	20
Jaylen	Direct and Indirect	Male	20
Justin	Direct and Indirect	Male	16
Miles	Direct and Indirect	Male	18
Cameron	Direct and Indirect	Male	19
Kyla	Indirect	Female	15
Dana	Indirect	Female	15
Jordan	Direct and Indirect	Male	16

Note. All youth participants identified as Black (n = 9). Ages ranged from 15 to 20 (M = 17.67, SD = 2.18).

## Data Availability

The data presented in this study are not publicly available due to privacy and ethical restrictions to protect participant confidentiality. De-identified excerpts from interviews and focus groups may be made available upon reasonable request from the corresponding author, with appropriate Institutional Review Board approval.

## Appendix A. Interview and Focus Group Topics

Interviews and focus groups explored the following domains. Youth participants reflected on their lived experiences, while clinicians were asked parallel questions about their observations of and work with their patient population.

1. Experiences of Community Violence

- Forms of exposure (e.g., witnessing violence, direct victimization, losing someone to violence).
- Immediate and long-term responses to violent events.

2. Neighborhood and Daily Context

- Neighborhood conditions and sense of safety.
- Experiences in places where participants attend school, work, or spend time.
- How community context shapes daily routines and decisions.

3. Emotional and Physical Responses

- Emotional reactions to violence and stress.
- Physical or somatic responses to trauma.
- How these responses show up in daily life.

4. Resilience and Meaning-Making

- How participants define and understand “resilience”.
- How others in their lives (family, peers, clinicians) define or interpret resilience.
- Ways youth demonstrate strength or adaptation.

5. Coping Strategies

- Strategies used to manage stress, trauma, or ongoing threat.
- What has been helpful versus unhelpful.
- Barriers to using preferred coping strategies.

6. Perceived Needs and Supports

- What participants believe they need to feel safe, supported, or emotionally well.
- What others (adults, providers, community members) think they need.
- Mismatches between perceived and actual needs.
- Recommendations for programs, services, or community supports.
